# Subcutaneous phaeohyphomycosis: ultrasound findings and histopathologic correlation

**DOI:** 10.1007/s00256-026-05287-z

**Published:** 2026-06-29

**Authors:** Alessandro Amorim Aita, Carolina Ávila de Almeida, Marcelo Volpatto Ortiz, Kay-Geert Hermann, Sara Severo Mendes da Paz, Rafael de Deus Moura, Clarissa Canella

**Affiliations:** 1 Hospital Universitário da Universidade Federal Do Piauí, Teresina, Piauí Brazil; 2 Hospital Universitário da Universidade Federal Do Rio de Janeiro, Rio de Janeiro, Brazil; 3 Hospital Universitário da Universidade Federal Fluminense, Niterói, Brazil; 4 Hospital Universitário Santa Terezinha-Joaçaba, Santa Catarina, Brazil; 5 Charité–Universitätsmedizin Berlin, Berlin, Germany

**Keywords:** Phaeohyphomycosis, Ultrasonography, Soft tissue infections, Subcutaneous lesions, Fungal infection

## Abstract

Subcutaneous phaeohyphomycosis is a rare opportunistic fungal infection caused by dematiaceous fungi and may clinically and radiologically mimic benign cystic or inflammatory soft tissue lesions. Imaging descriptions of this entity remain scarce. We report the case of a 60-year-old woman who presented with a slowly growing subcutaneous nodule. Ultrasound examination demonstrated a well-circumscribed hypoechoic pseudocystic lesion containing heterogeneous internal echoes and peripheral hypervascularity. Based on the imaging findings, the initial differential diagnosis included an epidermoid cyst and an inflammatory collection. Surgical excision followed by histopathological analysis revealed granulomatous inflammation containing pigmented septate fungal hyphae associated with a retained plant fragment (thorn), confirming the diagnosis of subcutaneous phaeohyphomycosis. Although the ultrasound appearance was not specific, it provided valuable characterization of lesion morphology, internal content, and vascularity, facilitating diagnostic assessment and guiding further management. This case is noteworthy because it illustrates the sonographic-pathologic correlation of a rare fungal infection associated with a retained vegetal foreign body and highlights imaging features that may increase awareness of this uncommon entity among radiologists, helping to reduce diagnostic delay and improve recognition of superficial fungal infections.

## Introduction

Subcutaneous mycoses comprise a heterogeneous group of infections involving the dermis and subcutaneous tissues, most commonly resulting from traumatic inoculation of environmental fungi. Among these, subcutaneous phaeohyphomycosis is a rare entity caused by dematiaceous (melanin-producing) fungi, including *Exophiala*, *Alternaria*, and *Bipolaris* species [1, 2].

Clinically, subcutaneous phaeohyphomycosis typically presents as a slowly enlarging, painless nodule or cyst-like lesion, most often affecting the extremities. Due to its nonspecific clinical appearance, it may mimic a variety of benign conditions, such as epidermoid cysts, ganglion, lipomas, or inflammatory collections, frequently leading to diagnostic delay. Histopathological analysis remains the gold standard for definitive diagnosis [2].

Although the dermatologic and microbiological features of phaeohyphomycosis are well established, its radiologic characterization remains limited. In particular, ultrasound has emerged as an important tool for the evaluation of superficial soft tissue lesions, enabling detailed assessment of lesion morphology, internal echotexture, vascularity, and its relationship with adjacent dermal and subcutaneous structures [3].

In this report, we describe the ultrasound findings of histologically confirmed subcutaneous phaeohyphomycosis and provide a focused review of the imaging features of subcutaneous fungal infections, emphasizing the role of ultrasound in the differential diagnosis of superficial soft tissue lesions.

## Case report

**Fig. 1:**
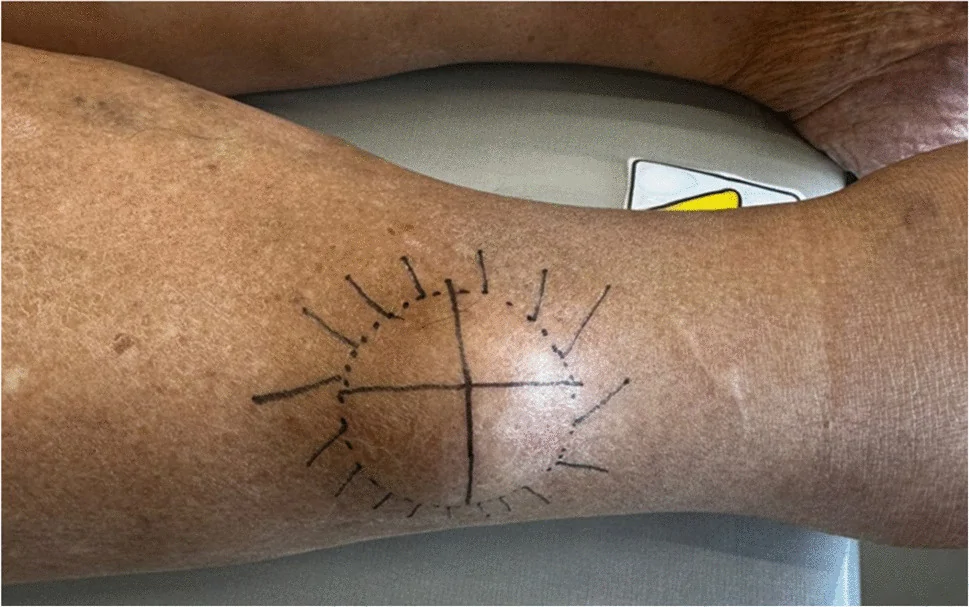
A 60-year-old woman with a clinical photograph of the lateral aspect of the leg demonstrating a well-defined subcutaneous bulging lesion without overlying skin discoloration. The lesion margins are outlined with a skin marker

A 60-year-old woman presented with a slowly enlarging subcutaneous nodule located in the anterior aspect of the distal third of the right leg. The lesion had progressively increased in size over several months and was painless, with no associated fever, systemic symptoms, or history of recent trauma. Clinically, it was suspected to represent an epidermoid cyst, abscess, or other benign soft tissue lesion. Physical examination revealed a firm, mobile subcutaneous mass without overlying skin ulceration or drainage (Fig. 1).

**Fig. 2:**
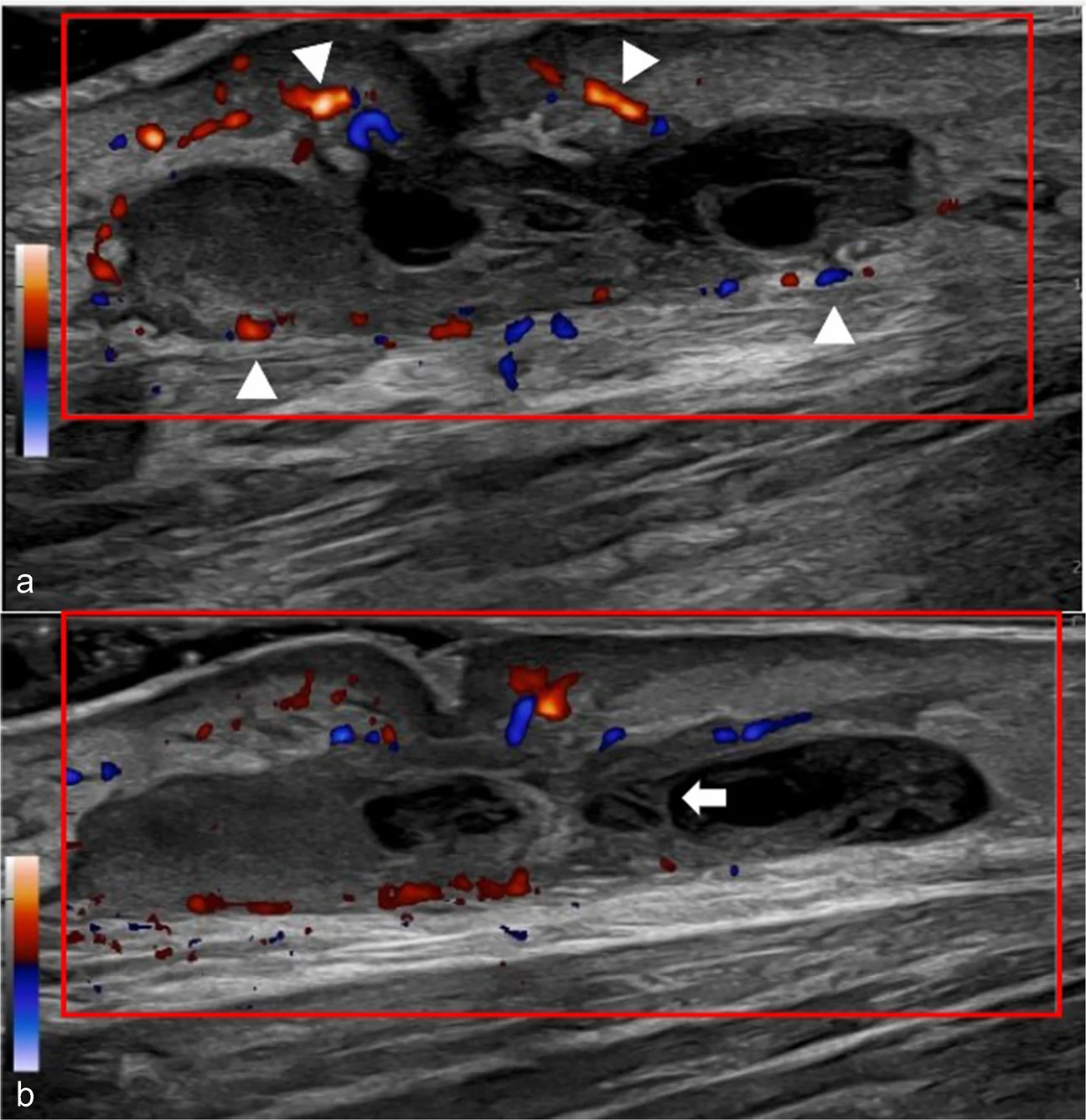
**a**, **b** Ultrasound images demonstrate a predominantly cystic subcutaneous lesion with thick internal septations (arrow) and peripheral vascularity on color Doppler imaging (arrowheads). The lesion is confined to the subcutaneous tissue, with preservation of the adjacent musculature

Ultrasound was performed using a linear-array transducer (18–22 MHz). Imaging demonstrated a well-circumscribed oval lesion within the superficial subcutaneous fat layer, without extension to the underlying fascia or musculature. The lesion exhibited a pseudocystic appearance, with predominantly hypoechoic content, heterogeneous internal echoes, and posterior acoustic enhancement (Fig. 2). Multiple internal echogenic debris-like foci were observed, resulting in a complex internal architecture rather than a simple cystic pattern. A thickened hypoechoic peripheral pseudocapsule was noted, suggesting a chronic inflammatory process. Color Doppler evaluation demonstrated mild peripheral hypervascularity within the wall and adjacent soft tissues, while no internal vascular flow was identified. No calcifications, sinus tracts, or foreign bodies were detected. Adjacent soft tissues showed mild inflammatory echogenicity, without evidence of fascial thickening or deep soft tissue involvement. Based on these imaging findings, the differential diagnosis included epidermoid cyst, inflamed epidermal inclusion cyst, localized abscess, foreign body granuloma, and fungal infection, including early mycetoma.

Given the progressive growth and indeterminate imaging features, as well as the superficial location and limited size of the lesion, complete surgical excision was performed without prior biopsy. Gross examination revealed a well-circumscribed cystic nodular lesion containing thick inflammatory material.

**Fig. 3:**
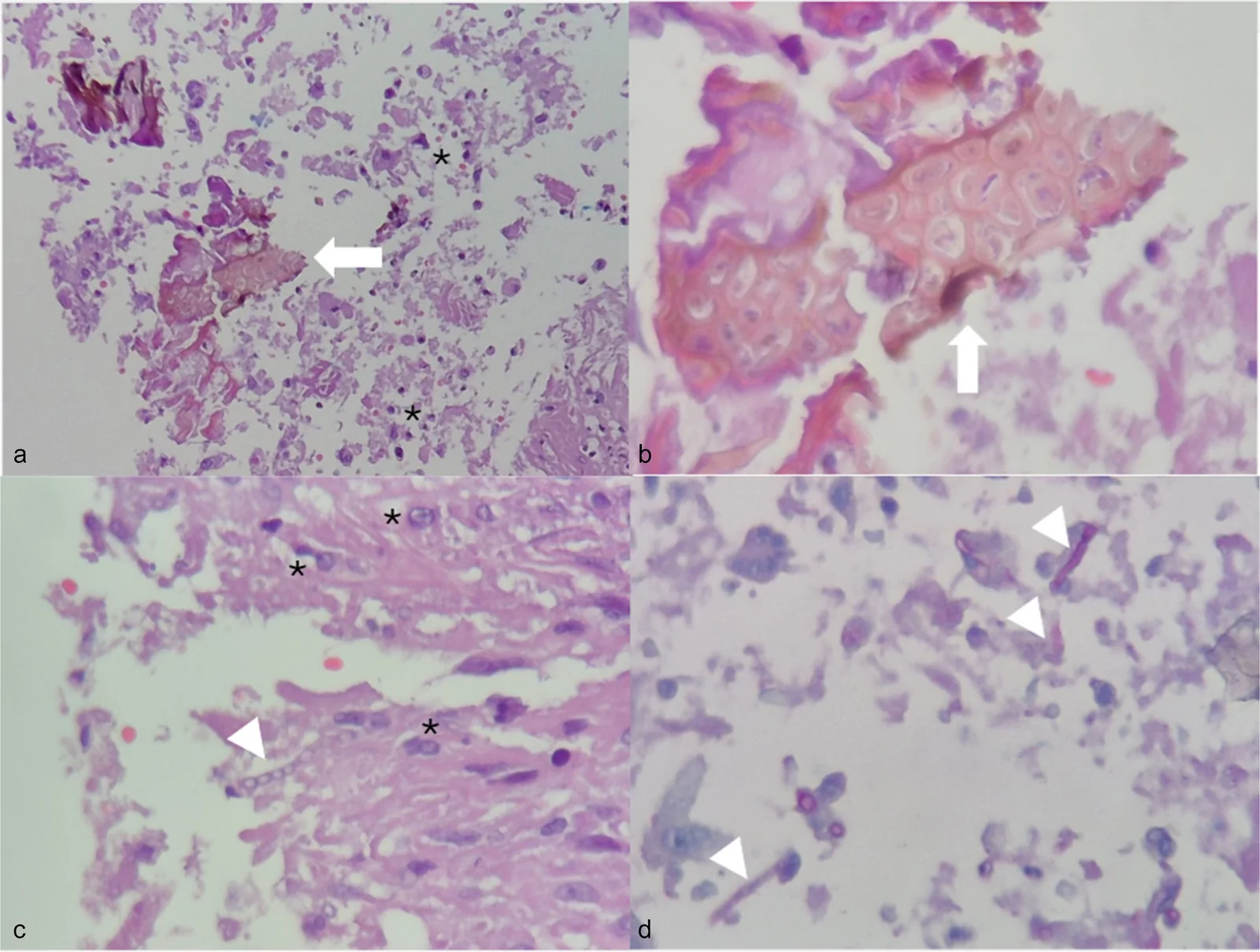
Histologic sections of the excised lesion. **a** Low-magnification photomicrograph demonstrating a plant fragment (thorn) (arrow) within an inflammatory background (asterisks) (H&E, × 40). **b** Higher-magnification detail of the plant fragment (arrow) (H&E, × 400). **c** Septate, subtly pigmented fungal hyphae (arrowheads) within the lesion (H&E, × 100). **d** Periodic acid–Schiff (PAS) staining highlighting the fungal hyphae (arrowheads) (× 100)

Histopathological analysis demonstrated a granulomatous inflammatory reaction involving the dermis and subcutaneous tissue, composed of epithelioid histiocytes, multinucleated giant cells, and lymphoplasmacytic infiltrates (Fig. 3). Foci of microabscess formation with neutrophilic aggregates were also identified. Within this inflammatory background, pigmented septate fungal hyphae were observed, focally showing short branching structures located within multinucleated giant cells.

Special stains, including periodic acid–Schiff (PAS) and Grocott methenamine silver (GMS), highlighted the fungal elements, confirming the presence of dematiaceous fungi. These findings established the diagnosis of subcutaneous phaeohyphomycosis. Immediate postoperative evolution was uneventful. However, long-term clinical follow-up data were not available because subsequent management occurred outside our institution.

## Discussion

Phaeohyphomycosis represents a heterogeneous group of infections caused by dematiaceous (melanin-producing) fungi, in which melanin within the fungal cell wall plays a central role in virulence by conferring resistance to host immune responses and oxidative stress [4, 5]. Although a wide spectrum of organisms has been implicated, including *Exophiala*, *Alternaria*, and *Bipolaris* species, the subcutaneous form is the most common clinical presentation, typically arising after traumatic inoculation of contaminated soil or plant material. While classically associated with immunosuppression, a significant proportion of cases occur in immunocompetent individuals, further contributing to its diagnostic challenge [4].

From a radiologic perspective, subcutaneous phaeohyphomycosis remains an underrecognized entity. The present case illustrates that the imaging appearance closely overlaps with far more common benign conditions, frequently leading to misclassification as an epidermoid cyst or inflammatory collection. In our patient, ultrasound demonstrated a well-circumscribed pseudocystic lesion with heterogeneous internal echoes, posterior acoustic enhancement, and peripheral hypervascularity, features that, although suggestive of a complex inflammatory process, are nonspecific [6]. This reinforces a critical limitation of imaging: While ultrasound provides exquisite spatial resolution for superficial lesions, it lacks specificity in distinguishing fungal infections from other cystic or inflammatory entities [3].

Nevertheless, subtle imaging features may raise diagnostic suspicion. The presence of internal echogenic debris within a pseudocystic lesion, combined with a thickened pseudocapsule and peripheral vascularity, should prompt consideration of an infectious etiology beyond a simple cyst. Importantly, the absence of the “dot-in-circle” sign helps differentiate phaeohyphomycosis from mycetoma, although this distinction is not always straightforward in early or atypical cases [7].

Despite these observations, definitive diagnosis relies on histopathological evaluation. The identification of granulomatous inflammation with multinucleated giant cells and pigmented septate hyphae remains the diagnostic hallmark, with PAS and Grocott methenamine silver stains facilitating confirmation. The absence of muriform bodies is a key feature distinguishing phaeohyphomycosis from chromoblastomycosis [3, 7]. In this case, histopathology was essential not only for diagnosis but also for excluding other infectious and noninfectious mimickers.

From a clinical standpoint, early recognition has direct therapeutic implications. Complete surgical excision is typically curative in localized disease, although adjunctive antifungal therapy may be required in selected cases. Failure to consider this diagnosis may result in incomplete treatment or inappropriate management, particularly when lesions are presumed to be benign cysts [3, 4].

Ultrasound is often the first-line imaging modality for superficial soft tissue lesions because of its excellent spatial resolution, dynamic capability, and ability to assess lesion vascularity in real time [8, 9]. In the present case, it confirmed the superficial subcutaneous location of the lesion, demonstrated its relationship to the adjacent fascia, and excluded deep muscular extension, findings that contributed directly to surgical planning. Doppler evaluation revealed peripheral hypervascularity, a feature that may favor an inflammatory or infectious process in contradistinction to a simple epidermoid cyst, although substantial imaging overlap persists. While not diagnostic, ultrasound provides valuable lesion characterization and may raise suspicion for atypical infectious etiologies among superficial pseudocystic lesions. Recognition of the imaging spectrum of subcutaneous phaeohyphomycosis, together with clinical and histopathological correlation, is important to avoid misdiagnosis and guide appropriate management [7].

## Conclusion

Subcutaneous phaeohyphomycosis is an uncommon fungal infection that may closely mimic benign cystic or inflammatory soft tissue lesions on imaging, representing a potential diagnostic pitfall. Ultrasound enables detailed characterization of superficial lesions and may reveal suggestive features, such as a pseudocystic appearance with internal echogenic debris and peripheral hypervascularity; however, these findings are not specific. Definitive diagnosis relies on histopathological confirmation. Therefore, radiologists should consider phaeohyphomycosis in the differential diagnosis of chronic or atypical subcutaneous lesions, particularly when imaging features are inconclusive. Early recognition and multidisciplinary correlation are essential to ensure accurate diagnosis and appropriate management.

## Data Availability

Data sharing is not applicable to this article as no datasets were generated or analyzed beyond those presented in the case report.
